# Direct Clinical Evidence Recommending the Use of Proteinase K or Dithiothreitol to Pretreat Sputum for Detection of SARS-CoV-2

**DOI:** 10.3389/fmed.2020.549860

**Published:** 2020-09-18

**Authors:** Jing Peng, Yanjun Lu, Juan Song, Bruce A. Vallance, Kevan Jacobson, Hong Bing Yu, Ziyong Sun

**Affiliations:** ^1^Department of Laboratory Medicine, Tongji Hospital, Tongji Medical College, Huazhong University of Science and Technology, Wuhan, China; ^2^Department of Gastroenterology & Endocrinology, Wuhan No. 9 Hospital, Wuhan, China; ^3^Department of Pediatrics, BC Children's Hospital Research Institute, University of British Columbia, Vancouver, BC, Canada

**Keywords:** sputum, detection of SARS-CoV-2, COVID-19, proteinase K (PK), DTT

## Abstract

One of the primary tools for diagnosing COVID-19 is the nucleic acid-based real-time reverse transcriptase-polymerase chain reaction (RT-PCR) test performed on respiratory specimens. The detection rate of SARS-CoV-2 in lower respiratory specimens (such as sputum) is higher than that for upper respiratory specimens (such as nasal and pharyngeal swabs). However, sputum specimens are usually quite viscous, requiring a homogenization process prior to nucleic acid (NA) extraction for RT-PCR. Sputum specimens from COVID-19 and non-COVID-19 patients were treated with four commonly used reagents—saline, N-acetyl-L-cysteine (NALC), proteinase K (PK), and dithiothreitol (DTT), prior to NA extraction. These reagents were then compared for their performance in diagnosing COVID-19 in real clinical practice. The detection rate of SARS-CoV-2 in PK- or DTT-treated sputum was comparable, and higher than that in sputum treated with NALC or saline. While there was a 4.8% (1/21) false negative rate for the PK- and DTT-treated sputum, neither treatment showed any false positive cases among patients with non-COVID diseases. Moreover, sputum pretreated with saline, NALC, PK or DTT showed higher detection rates of SARS-CoV-2 as compared to pharyngeal swabs. Taken together, we provide direct evidence recommending the use of PK or DTT to pretreat sputum samples to facilitate SARS-CoV-2 detection by clinical laboratories. Moreover, our methods should help to standardize the procedure of processing sputum specimens and improve the ability to detect SARS-CoV-2 in these samples.

Coronavirus disease 2019 (COVID-19) outbreak caused by SARS-Cov-2 was declared a global pandemic on Mar 11, 2020. One of the primary tools for diagnosing COVID-19 is the nucleic acid-based, real-time reverse transcriptase-polymerase chain reaction (RT-PCR) test, performed on respiratory specimens. The detection rate of SARS-CoV-2 in lower respiratory specimens (such as sputum) is higher than that obtained with upper respiratory specimens (such as nasal and pharyngeal swabs) (1). However, sputum samples often contain a large amount of mucus and are viscous, resulting in the trap of virus containing cell components within the mucus. This will prevent nuclei acid (NA) extraction reagents from accessing to these components, leading to low yield of RNA. Without being homogenized sufficiently, sputum samples can have multiple adverse effects, such as introducing cross-contamination to the automatic nucleic acid extraction instrument, and causing pipetting errors, clot formation, or failed amplification (2). Surprisingly, a number of kits used for detecting SARS-CoV-2 in sputum lack specific instructions on how to homogenize sputum samples before NA extraction.

Several reagents have been used to homogenize sputum samples, such as proteinase K (PK), dithiothreitol (DTT), and N-acetyl-L-cysteine (NALC). PK is a stable serine alkaline protease, and has broad substrate specificity (2, 3). It is often used to digest abundant proteins present in sputum samples, and preferentially degrades ester and peptide bonds next to the C-termini of hydrophobic, sulfuric, or aromatic amino acids. This digestion process inactivates nucleases that could degrade DNA or RNA during isolation and purification procedures. DTT has a very low redox potential, and is able to quantitatively reduce disulfide bonds and maintain monothiol in a reduced state. As a source of the reactive sulfhydryl groups, NALC is mucolytic (4). It prevents the formation of intramolecular and intermolecular disulfide bonds in sputum samples. By disrupting disulfide bonds, both DTT and NALC are widely used to liquefy mucus (5, 6). The proper homogenization and liquefaction of mucus by PK, DTT, and NALC will help to remove substances that inhibit amplification, as well as increase the yield of extracted RNA. This will ultimately improve the detection of virus RNA by RT-PCR. Using spiked sputum samples, a previous study showed that PK-DNase method was ideal for homogenizing sputum samples prior to RT-PCR for the detection of Middle East respiratory syndrome coronavirus (MERS-CoV) (7). Sputum appeared to be a good clinical specimen in patients at the early stage of SARS infection (8), and it also outperformed nasal/pharyngeal swabs in detecting other respiratory viruses, such as respiratory syncytial virus, parainfluenza virus, and human metapneumovirus (9). More recently, SARS-CoV-2 is shown to be more readily detected in sputum samples than in throat swabs of convalescent COVID-19 patients (10). Despite these findings, clinical assessment is lacking regarding how sputum should be pretreated for its best performance in diagnosing SARS-CoV-2 infections. To address this, we treated clinical sputum specimens with four commonly used reagents—saline, NALC, PK, and DTT, prior to NA extraction, and compared their performance in diagnosing COVID-19 in real practice.

## Methods

### Sputum Collection

A total of 68 sputum specimens were collected from adult patients admitted to Tongji Hospital, Huazhong University of Science and Technology (Wuhan, China) between Jan 28 and Mar 2, 2020. Of these patients, 21 were diagnosed as SARS-CoV-2 positive (SARS-CoV-2^+^) based on clinical symptoms (fever, cough, and dyspnea), computed tomography (CT) and/or RT-PCR results from pharyngeal swabs. The remaining 47 patients had diseases unrelated to COVID-19. This study was approved by the ethics committee of Tongji Hospital (TJ-C2030).

### Sputum Treatment

All sputum specimens were repeatedly pipetted up and down every 5 min, and vortexed for 30 min. Each sputum specimen was aliquoted into four Eppendorf tubes (500 μl per tube), followed by the addition of 500 μl of saline, NALC (0.5 g/100 ml, Sinopharm Chemical Reagent Co. Ltd, freshly made), PK (1 g/l, TianLong Science and Technology Co., Ltd., Xi'an, China), and DTT (Sputasol, Oxoid Microbiological Products) into each tube. Samples were kept upright at room temperature for 30 min, and pipetted up and down once at the 15 min interval, until completely liquefied.

### RNA Extraction and RT-PCR Test

Liquefied sputum samples were centrifuged, and the supernatants (250 μl) were used for RNA extraction on a fully automated nucleic acid extraction system 9600E, using the NA extraction kit from Tianlong Science & Technology (TianLong Science and Technology Co., Ltd., Xi'an, China). The RT-PCR was performed with a SARS-CoV-2 nucleic acid detection kit according to the manufacturer's instructions (Da'an Gene Co., Ltd. of Sun Yat-Sen University, Guangzhou, China, approved by National Medical Products Administration). This kit detects *ORF1ab* and N genes from SARS-CoV-2. Samples with cycle threshold values (Ct-values) for both genes ≤ 40, or a Ct-value of only one gene ≤ 40, repeated twice, were defined as SARS-CoV-2^+^. Samples with Ct-values for both genes > 40, or not showing an amplification curve for either gene, were defined as SARS-CoV-2 negative.

## Results

**Figure 1:**
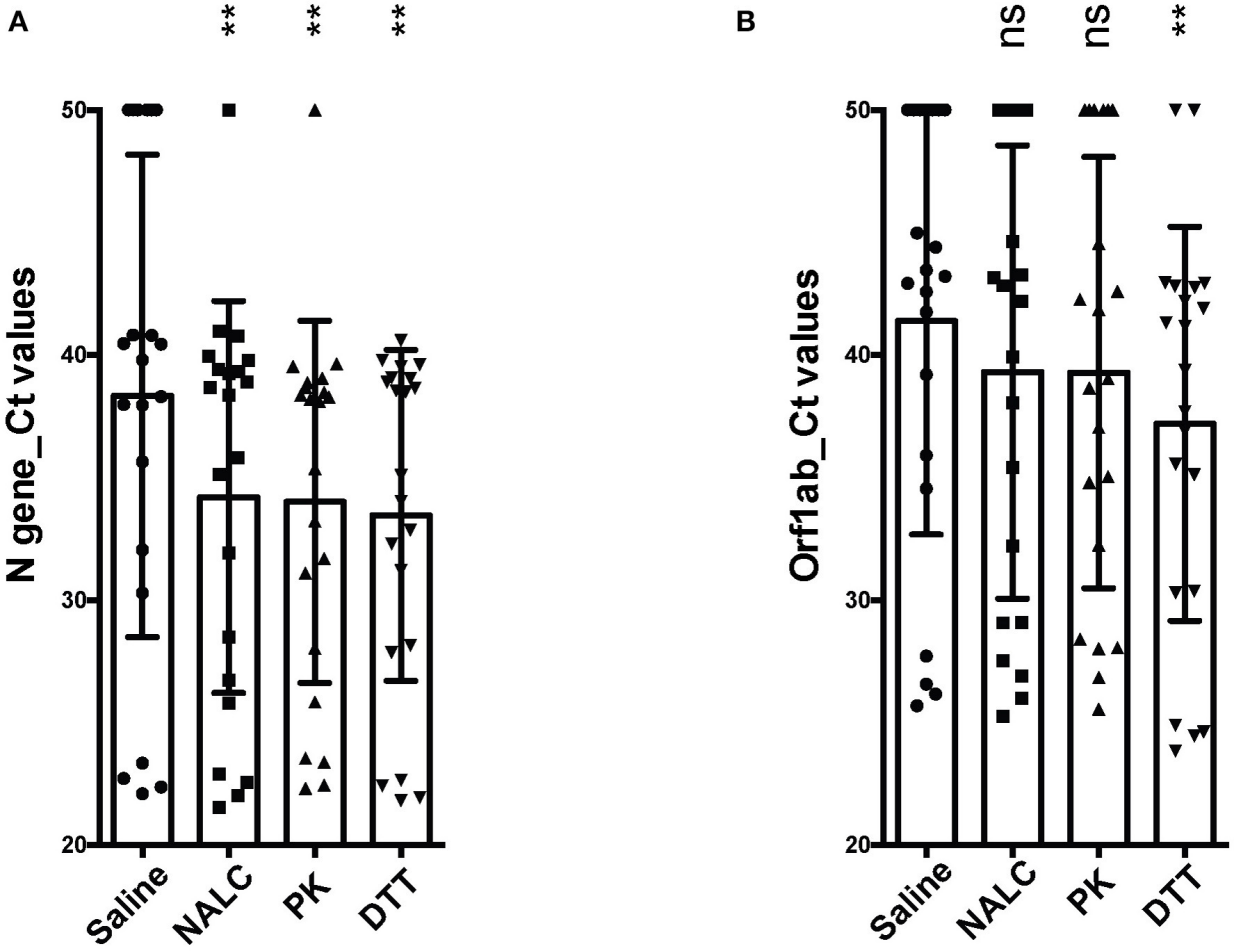
Comparison of different pretreatments for detection of SARS-CoV-2 from sputum. **(A)** Ct-values of the N gene from clinical sputum specimens under different treatments. **(B)** Ct-values of the *ORF1ab* gene from clinical sputum specimens under different treatments. Statistical differences were calculated using one-way ANOVA with *post-hoc* turkey multiple comparison test. For clinical sputum specimens showing no amplication curves, Ct-values of 50 were arbitrarily assigned to calculate statistical differences. **p < 0.01; ns, not significant. NALC, N-Acetyl-L-Cysteine; PK, Proteinase K; DTT, Dithiothreitol.

With the sputum samples collected from the 47 patients having non-COVID-19 diseases, no amplification curves were observed for either the *ORF1ab* or N gene under any treatment conditions (saline, NALC, PK, and DTT), suggesting no SARS-CoV-2 in these samples. In contrast, RT-PCR tests of all the sputum samples from the SARS-CoV-2^+^ patients showed Ct-values for both genes <40, or Ct-values of one gene <40 (repeated twice), under at least one treatment condition. Ct-values of the N gene from saline-, NALC-, PK-, and DTT-pretreated sputum samples were 38.3 ± 9.8, 34.2 ± 8.0, 34.0 ± 7.4, 33.4 ± 6.8, respectively, with the NALC-, PK-, and DTT-pretreated groups significantly lower than the saline-treated group (Figure 1A). Ct-values of the *ORF1ab* gene from these treatments were 41.4 ± 8.7, 39.3 ± 9.2, 39.3 ± 8.8, 36.9 ± 8.0, respectively, with the DTT-pretreated group significantly different from the saline-treated group (Figure 1B).

According to the positive criteria described in the methods section, pretreatment of sputum samples with NALC, PK, and DTT increased the detection of SARS-CoV-2^+^ cases to 85.7% (18/21), 95.2% (20/21), and 95.2% (20/21), respectively, as compared to the 52.4% (11/21) obtained with saline pretreated sputum (see Table 1). Of the 8 mucopurulent sputum samples, only 1 was found to be SARS-CoV-2 negative when treated with PK or DTT. Notably, the amount of sputa required for the detection of SARS-CoV-2 by NALC, PK, or DTT treatments was as low as 0.2 ml.

**Table 1:** Summary of sputum test results and sputum characteristics.

**Patient ID**	**Treatment**					**Sputum characteristics**		
	**Saline**	**NALC**	**PK**	**DTT**	**Swabs**	**Viscosity**	**Appearance**	**Amount (ml)**
1	–	+	+	+	NA	Moderate	Mucopurulent	2.5
2	–	–	+	+	NA	High	Mucopurulent	1.6
3	–	+	+	+	–	Moderate	Mucoid	2
4	–	+	+	+	–	Mild	Mucoid	1.5
5	–	+	+	+	NA	High	Mucoid	8
6	+	+	+	+	+	Moderate	Mucopurulent	6
7	+	+	+	+	+	Mild	Blood-tinged	1.2
8	+	+	+	+	+	Moderate	Blood-tinged	1.4
9	+	–	+	+	–	Mild	Mucoid	0.8
10	–	+	+	+	–	High	Mucopurulent	0.4
11	–	+	+	+	–	Moderate	Mucoid	1.5
12	–	+	+	+	NA	Mild	Mucoid	3
13	+	+	+	+	NA	Mild	Mucoid	4
14	+	+	+	+	–	High	Mucopurulent	0.5
15	+	+	+	+	NA	Mild	Mucoid	16
16	+	+	+	–	–	Moderate	Mucopurulent	1
17	–	+	+	+	+	High	Mucopurulent	0.2
18	+	+	+	+	–	High	Mucopurulent	2.5
19	+	+	+	+	+	Moderate	Blood-tinged	1.5
20	–	–	–	+	–	Moderate	Mucopurulent	1.2
21	+	+	+	+	+	High	Blood-tinged	2

*A total of 21 sputum specimens were obtained from SARS-CoV-2 positive patients. Saline, NALC, PK, and DTT treatments of sputum were able to detect 52.4% (11/21), 85.7% (18/21), 95.2% (20/21), and 95.2% (20/21) of SARS-CoV-2^+^ cases, respectively. Pharyngeal swabs were able to detect 40.0% (6/15) of SARS-CoV-2 positive cases. NA, not available*.

Of the 21 SARS-CoV-2^+^ patients, 15 also had pharyngeal swabs collected within 1 day of sputum collection (i.e., when sputum was used for RT-PCR test), with only 40% (6/15) of these patients identified as SARS-CoV-2^+^ based on testing the swabs by RT-PCR.

## Discussion

Based on previous experience with other respiratory viruses, the USA CDC has recommended the use of DTT to pretreat sputum for the detection of SARS-CoV-2. However, there is a lack of direct clinical evidence supporting this suggestion or the use of other chemicals to pretreat sputum prior to performing RT-PCR tests for SARS-CoV-2.

We examined how different pretreatments affected SARS-CoV-2 detection from sputum specimens. Our RT-PCR assay showed a higher sensitivity for detecting the N gene, similar to that reported by others (11, 12). This likely results from the relative amplification differences between the *ORF1ab* or N genes, as the N gene has a much higher level of subgenomic mRNA (13). The homogenization of sputum by PK and DTT appeared to be very thorough, as these pre-treatments increased the positive rates by ~32.8% as compared to saline-treated sputum. Similar to our report, the detection of human avian influenza A (H7N9) virus from sputum samples is also improved by PK and DTT pretreatment (14). Pretreating sputum samples with NALC also improved the detection of SARS-CoV-2^+^ cases, with freshly made NALC improving the detection rate by 15% as compared to 1-day-old NALC (data not shown). However, the detection rate of SARS-CoV-2 in NALC-treated sputum was lower than that obtained with PK- or DTT-treated sputum. One plausible reason for this could be that PK and DTT are able to digest mucous protein more completely than NALC, resulting in increased concentration of extracted RNA. Supporting this, DTT (a dithiol having two redox-active cysteine residues) is more effective than NALC (a monothiol) in reducing sputum elasticity (15, 16).

Sputum pretreated with saline, NALC, PK, or DTT showed a higher detection rate than when assessing pharyngeal swabs, a finding similar to that outlined in two recent reports (1, 17). While there was only a 4.8% (1/21) false negative rate for the PK- or DTT-treated sputum samples, neither treatment caused any false positive cases among patients with non-COVID-19 diseases. These data suggest that testing sputum may be a preferred approach to diagnosing COVID-19, as well as differentiating SARS-CoV-2 from other prevalent viral infections that cause similar symptoms (18). Such approaches could potentially decrease the pressure on critical care resources in hospitals where multiple pharyngeal swabs are often required to rule in/out COVID-19. Currently, many point-of-care tests (POCT) have already been developed for SARS-CoV-2 detection. These POCT tests will not only significantly reduce time of testing, but also help to optimize clinical management and increase patient satisfaction (19). While these tests are most likely effective with nasal/pharyngeal swabs or aspirates, it may not work well with non-homogenized sputum. Our findings thus provide a very promising scenario whereby sputum specimens pretreated with PK or DTT, in conjunction with these POCT tests, could significantly increase the accuracy of diagnosing COVID-19 as well as provide rapid diagnosis.

This study was limited to the single collection of sputum from a small number of SARS-CoV-2^+^ inpatients with moderate to severe infections. Further studies should include more patients, particularly those with mild infections, as well as collect sputum at least twice from each patient. Also, in some cases, there is a limited availability of sputum from COVID-19 patients. In a study with a larger population, ~33% of COVID-19 patients produced sputum (20), and the majority of COVID-19 patients may produce no or very limited amount of sputum. However, since the amount of sputum required for this assay is as low as 0.2 ml, we anticipate that pretreating sputum samples with PK or DTT will facilitate SARS-CoV-2 detection from patients who fail to produce abundant sputum.

## Conclusion

In summary, while it is known that sputum samples usually outperform nasal/pharyngeal swabs in detecting respiratory viruses, including SARS-CoV-2, the performance of sputum samples in the diagnosis of COVID-19 can be further affected by the way how sputum was pretreated. We provide direct clinical evidence recommending the use of PK or DTT to pretreat sputum to facilitate SARS-CoV-2 detection in clinical laboratories. This recommendation could be further confirmed with more diverse clinical samples, ultimately improving the procedure of processing sputum specimens for their best performance in detecting SARS-CoV-2.

## Data Availability Statement

The raw data supporting the conclusions of this article will be made available by the authors, without undue reservation.

## Ethics Statement

This study was approved by the ethics committee of Tongji Hospital (TJ-C2030). Written informed consent for participation was not required for this study in accordance with the national legislation and the institutional requirements.

## Author Contributions

JP, HY, and ZS had full access to all of the data in the study and take responsibility for the integrity of the data. JP, YL, JS, HY, and ZS: concept and design, acquisition, analysis, or interpretation of data. JP and HY: drafting of the manuscript. BV and KJ: critical revision of the manuscript for important intellectual content. HY: statistical analysis. ZS: obtained funding. HY and ZS: supervision. All authors contributed to the article and approved the submitted version.

## Conflict of Interest

The authors declare that the research was conducted in the absence of any commercial or financial relationships that could be construed as a potential conflict of interest.
